# Mammalian Genomic Manipulation with Orthogonal Bxb1 DNA Recombinase Sites for the Functional Characterization of Protein Variants

**DOI:** 10.1021/acssynbio.3c00355

**Published:** 2023-11-03

**Authors:** Sarah M. Roelle, Nisha D. Kamath, Kenneth A. Matreyek

**Affiliations:** Department of Pathology, Case Western Reserve University School of Medicine, Cleveland, Ohio 44106, United States

**Keywords:** Bxb1, recombinase, landing pad, GCaMP, HIV-1 Vif, Apobec3

## Abstract

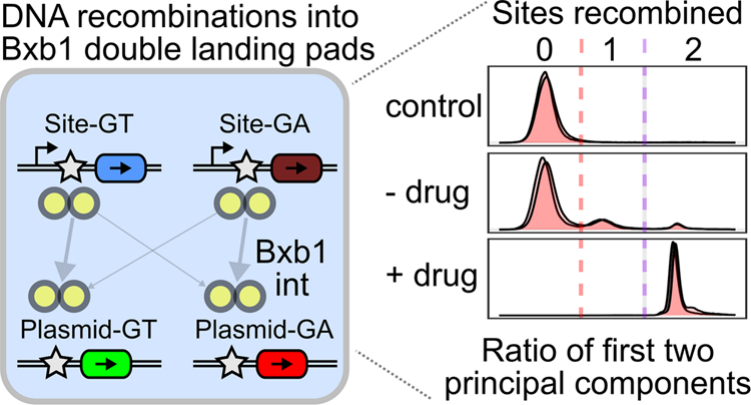

The Bxb1 bacteriophage serine DNA recombinase is an efficient tool for engineering recombinant DNA into the genomes of cultured cells. Generally, a single engineered “landing pad” site is introduced into the cell genome, permitting the integration of transgenic circuits or libraries of transgene variants. While sufficient for many studies, the extent of genetic manipulation possible with a single recombinase site is limiting and insufficient for more complex cell-based assays. Here, we harnessed two orthogonal Bxb1 recombinase sites to enable alternative avenues for using mammalian synthetic biology to characterize transgenic protein variants. By designing plasmids flanked by a second pair of auxiliary recombination sites, we demonstrate that we can avoid the genomic integration of undesirable bacterial DNA elements using the same starting cells engineered for whole-plasmid integration. We also created “double landing pad” cells simultaneously harboring two orthogonal Bxb1 recombinase sites at separate genomic loci, allowing complex cell-based genetic assays. Integration of a genetically encoded calcium indicator allowed for the real-time monitoring of intracellular calcium signaling dynamics, including kinetic perturbations that occur upon overexpression of the wild-type or variant version of the calcium signaling relay protein STIM1. A panel of missense mutants of the HIV-1 accessory protein Vif was paired with various paralogs within the human Apobec3 innate immune protein family to identify combinations capable or incapable of interacting within cells. These cells allow transgenic protein variant libraries to be readily paired with assay-specific protein partners or biosensors, enabling new functional readouts for large-scale genetic assays for protein function.

## Introduction

Site-specific DNA recombinases play an important role in mammalian cell engineering and synthetic biology, as they permit the efficient, precise, and stable integration of large transgenic DNA sequences into the cell genome.^1^ Tyrosine recombinases such as Cre or Flp are particularly useful for genetic excision reactions, such as when generating conditional knockout mice, but can be inefficient for integration reactions due to their reversibility.^2^ In contrast, genetic integration reactions with serine recombinases such as PhiC31 and Bxb1 are irreversible in the absence of recombinase directionality factors, such as Bxb1 gp47.^3^ The Bxb1 DNA recombinase has been particularly effective due to its high rates of enzymatic activity, irreversibility, and the lack of confounding pseudosites in the human cell genome.^4,5^ Bxb1 has thus become the site-specific DNA recombinase of choice for various new biotechnologies, including STRAIGHT-IN^6^ and PASTE.^7^

These characteristics have accelerated the adoption of Bxb1-based transgenic expression systems with multiplex assays of variant effect (MAVE), such as deep mutational scanning (DMS), as its efficiency allows the testing of large libraries of protein variants *en masse*. Here, each cell possesses a single recombinase “landing pad” site preceded by a synthetic promoter, which can integrate and express a single variant from a library of transfected promoterless plasmids.^8,9^ For Bxb1, the recombinase sites are termed attP and attB, which recognize each other and permit exogenous DNA integration in engineered mammalian cell genomes, much like how the Bxb1 bacteriophage integrates its DNA into a bacterial host’s genome. This technology enables a wide range of potential applications, including the study of the impacts of missense variants on protein steady-state abundance for informing cancer or clinical genetics,^10^ identification of potential resistance mutations against chemotherapeutics,^11^ or engineering improved biosensors.^12^

Despite their general utility, single-site landing pad cells exhibit limitations. Complex assay systems may require insertion of over a half-dozen transgenic elements, which require large plasmid molecules that must be sequence-confirmed after every modification to ensure the absence of unintended mutations. In another case, it may be informative to test a variant library under slightly different genetic conditions, and having an additional site for cell engineering would allow the original variant library to be kept unchanged. Both situations would be aided by having a second, orthogonal “landing pad” site capable of accommodating additional transgenic elements alongside the variant library introduced into the original landing pad site. By having single copies of two orthogonal landing pad sites, it should be possible to gain this additional engineering flexibility while maintaining the strict genotype–phenotype links and high replicate variant homogeneities critical for multiplex genetic assays.

In this work, we demonstrate the advanced genetic assays that are possible from having two orthogonal Bxb1 recombinase sites: one with a central “GA” dinucleotide sequence^13^ and the other the more traditionally used central “GT” dinucleotides. We confirm that these sequences are orthogonal, with matched recombination occurring 100-fold more efficiently than mismatched recombination. This enabled the creation of integration plasmids capable of automatically excising undesired bacterial elements once they were introduced into cultured mammalian cells. We also created “double landing pad” cells capable of reproducibly integrating single copies of two unique plasmid molecules within the same cell, enabling the pairing of protein variant libraries with fluorescent biosensors or various physically interacting intracellular protein partners. Finally, we show that other Bxb1 central dinucleotides can be incorporated with equivalent levels of orthogonality to create even more flexible and orthogonal transgenic systems. These advancements in mammalian synthetic biology will enable previously unachievable experiments with protein variant libraries in cell-based assays.

## Results

**Figure 1:**
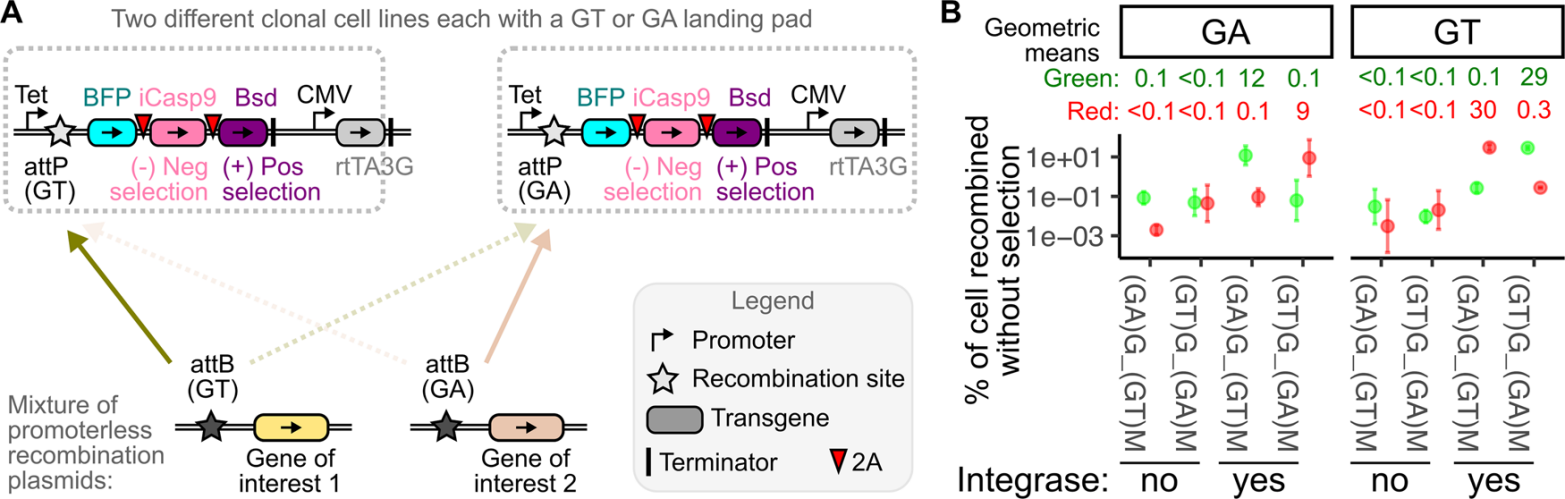
Orthogonality of Bxb1 GT and GA central dinucleotide recombination sites. (A) Construct schematics for testing orthogonality of recombination into the landing pad. (B) Experimentally assessed orthogonality of GA- or GT central dinucleotide Bxb1 attP and attB pairs. Labels at the top denote the central dinucleotide in the genomically integrated landing pad attP site. attB plasmid key: (GA)G denotes GFP behind a GA dinucleotide, (GA)M denotes mCherry behind a GA dinucleotide, (GT)G denotes GFP behind a GT dinucleotide, and (GT)M denotes mCherry behind a GT dinucleotide. Circles denote the geometric mean value, while error bars denote 95% confidence intervals, calculated from three replicate experiments, performed in the absence of any positive or negative selection. Abbreviations: Tet, tetracycline-inducible promoter; BFP, blue fluorescent protein; iCasp9, AP1903-inducible caspase 9; Bsd, blasticidin S deaminase; CMV, Cytomegalovirus immediate early promoter; and rtTA3G, reverse tet transactivator third generation.

We first established the orthogonality of the GA and GT dinucleotide Bxb1 recombination sites with our own engineered cells. To do this, we created two clonal landing pad cell lines: one with a central GT dinucleotide and the other with a central GA dinucleotide. Each cell line was transfected with an equimolar mixture of attB recombination plasmids encoding EGFP or mCherry (Figure 1A). In the original mixture, EGFP was behind the GA attB site (“[GA]G”), while mCherry was behind the GT attB site (“[GT]M”). A separate plasmid mixture was made with the orientations swapped (“[GA]M” and “[GT]G”). Five or more days after transfection, the number of green or red cells was assessed with flow cytometry.

Only the fluorophore with the attB site matched to the landing pad attP site exhibited clear recombination, modifying ∼10 to 30% of the cells of the well without any selective agents added (Figure 1B). Despite being present in equimolar amounts, the fluorophore behind the mismatched attB site resulted in 100-fold fewer fluorescent cells than the matched fluorophore. This level of fluorescence was at or slightly above the background frequency of fluorescent cells observed when the Bxb1 integrase expression plasmid was omitted.

The orthogonality of the GA and GT sites yielded a new avenue for removing unwanted bacterial sequence from the integrated DNA. Our existing Bxb1 landing pad design harnesses a single genomic recombination site, but this results in the integration of the entire plasmid molecule, including the bacterial origin of replication and various antibiotic resistance cassettes for propagating the plasmid DNA in bacteria.^8,9^ In many cases, the presence of the bacterially derived sequences is inconsequential for downstream assays, although there are exceptions, such as highly sensitive β-lactamase CCF2-dye conversion assays,^14^ which can be muddied by even minute amounts of enzymatic activity stemming from an integrated ampicillin resistance gene.

Traditional engineering schemes have relied upon recombinase-mediated cassette exchange (RMCE) to swap the new transgenic elements in place of the old ones while avoiding integration of the bacterially derived sequences. While orthogonal recombination sequences can be used for RMCE with Cre or Flp recombinases, these tyrosine recombinases lack the efficiencies necessary for large-scale multiplex genetic experiments that potentially require millions of cells to be independently recombined within a given experiment. Previous designs with serine recombinases, such as dual integrase cassette exchange (DICE),^15^ use two different bacteriophage recombination sites flanking a DNA cassette to be exchanged. Unfortunately, DICE requires the simultaneous overexpression of both the Bxb1 and comparatively less efficient and less specific PhiC31 recombinase enzymes and successful cassette exchange requires recombination at both sites (the product of two potentially low-probability events). Thus, the majority of transfected cells likely exhibit recombination intermediates, where only one site has recombined, generating heterogeneity in the resulting cell population. Furthermore, RMCE formats, including DICE, require genomically integrated landing pads harboring the same recombination sites in the same order as the plasmids to be recombined and are thus inherently incompatible with existing cells that were engineered to harbor only a single recombination site.

**Figure 2:**
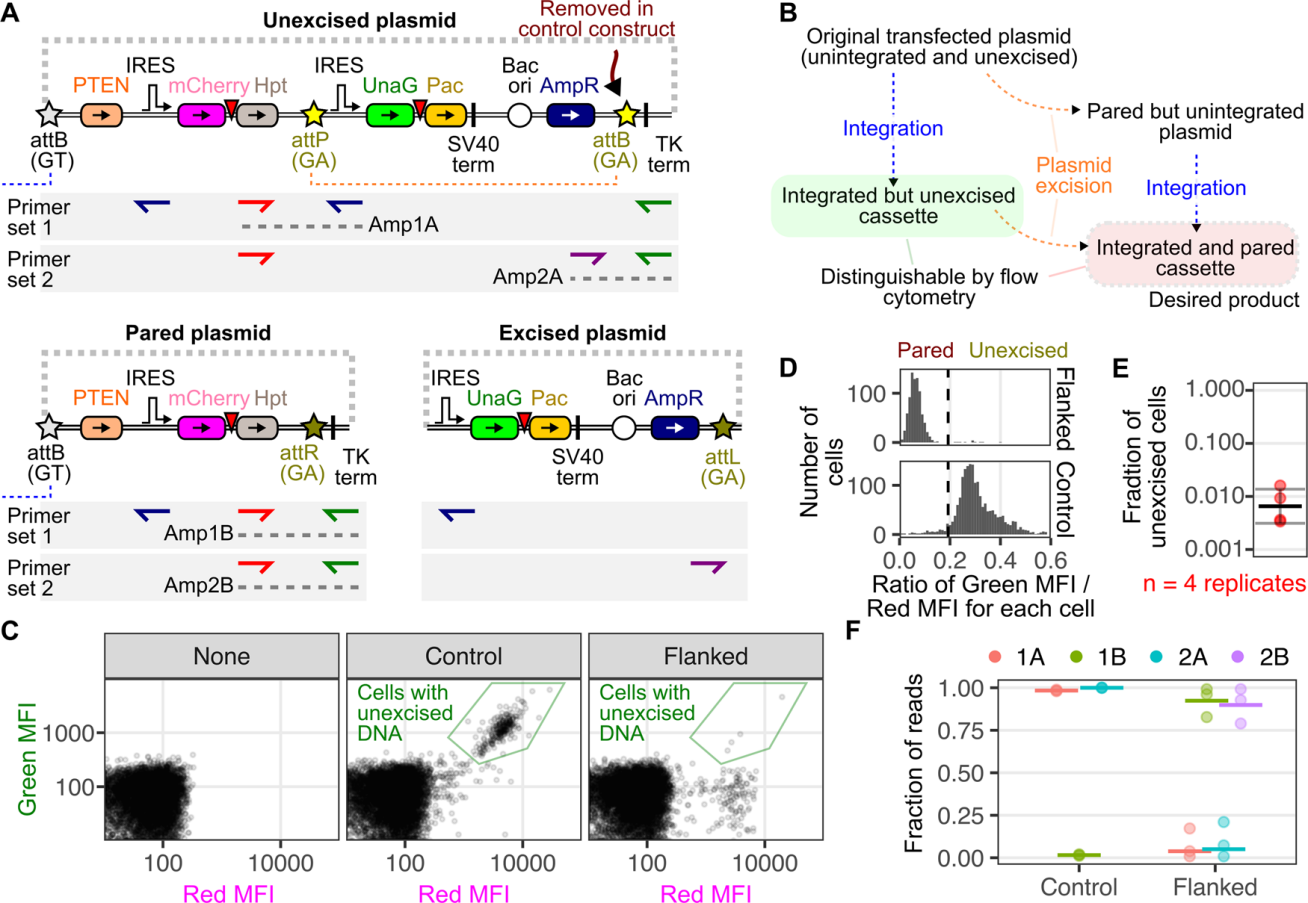
Excision of undesired bacterial sequence using flanking GA recombination sites. (A) Schematic of the recombination plasmid generated to test GA site-based excision of bacterial sequence. The top panel shows the construct before GA site recombination, while the bottom panels show the two products expected after recombination occurs. The brown arrow indicates the recombination site that was removed to make the control construct that is incapable of excision. (B) Schematic illustration of possible intermediate and final products, when the excision occurs either as a plasmid or as integrated genomic DNA. (C) Example flow cytometry plots showing the patterns of green and red fluorescence observed with the GA site flanked construct, or the control construct where paring of the plasmid cannot occur as one recombination site was deleted. (D) Example green/red ratiometric histograms for the flanked and control constructs, with the threshold value above which the highest 95% of control cell values measured shown as the dotted line. A constant value of 250 was added to all green MFI values to render all values positive. (E) Upon transfection of flanked construct, the fraction of red cells that were also green, indicative of failed excision. Red points are individual replicates, and the black bar is the geometric mean of four replicate experiments. (F) Fraction of Illumina sequencing reads corresponding to each amplicon product, with different primer sets, for cells transfected with the two constructs. The primer-binding sites and expected amplicons are shown in the schematic in panel A. The cells were selected with hygromycin prior to genomic DNA extraction and PCR. Abbreviations: PTEN, phosphatase and tensin homologue gene as transgenic cargo; mCherry, red fluorescent protein; IRES, internal ribosome entry site; Hpt, hygromycin phosphotransferase gene; UnaG, green fluorescent protein derived from eel; Pac, puromycin *N*-acetyltransferase; Bac ori, bacterial origin of replication; AmpR, ampicillin resistance gene; SV40 term, transcriptional terminator from simian virus 40; and TKterm, transcriptional terminator from herpes simplex virus thymidine kinase.

We melded these concepts and used orthogonal Bxb1 sites for a bacterial sequence excision approach. Here, integration into the genomic landing pad site is still driven by recombination of the GT dinucleotide Bxb1 recombination site, but the undesired bacterial sequence is flanked by a pair of GA dinucleotide attP and attB sites (Figure 2A). Thus, the Bxb1 recombinase enzyme is responsible for both genetically integrating the desired transgenic sequence (Figure 2B and Supporting Information Figure S1, blue lines) and removing the undesired sequence (Figure 2B and Supporting Information Figure S1, orange lines). Successful DNA excision yields an extrachromosomal bacterial DNA sequence that will eventually become degraded, while the now pared desired transgenic sequence becomes stably retained through genomic integration.

To test this approach, we created a specialized recombination plasmid encoding a green fluorescent protein, UnaG, adjacent to the bacterial sequence. The UnaG transgene is still encoded on the same mRNA as mCherry but is separated by a GA attP site. Thus, landing pad cells where the entire unexcised plasmid has integrated should exhibit both red and green fluorescence, whereas cells where the bacterial portion has excised should only exhibit red fluorescence (Figure 2B and Supporting Information Figure S2). To test this concept, we created a control construct incapable of excision, where the GA attB site was deleted (Figure 2A).

Upon transfection into landing pad cells, the control construct yielded a large fraction of red and green cells (Figure 2C, middle). In contrast, transfection of the GA flanked construct yielded mostly red fluorescent cells, with a minor fraction being both red and green (Figure 2C, right). The fraction of unexcised cells could be quantitated by taking the ratio of green-to-red mean fluorescence intensity per cell and determining the number of cells in the flanked construct sample that exhibited a value within the distribution for the control sample (Figure 2D). Replicate experiments with the flanked construct showed that only ∼1% of red fluorescent cells also exhibited green fluorescence, indicating that only a minor fraction of cells harbored unexcised bacterial sequences (Figure 2D,E). To confirm this, we designed amplicons for both excised and unexcised plasmid DNA, and the relative proportions of each product were counted with high-throughput DNA sequencing. Consistent with our flow cytometry results, the vast majority of amplified products were excised, with 5% or less of the amplified DNA corresponding to the unexcised plasmid (Figure 2F). The DNA sequencing data are unable to distinguish integrated from unintegrated transfected plasmid, likely accounting for the numerical discrepancies between the two assays.

**Figure 3:**
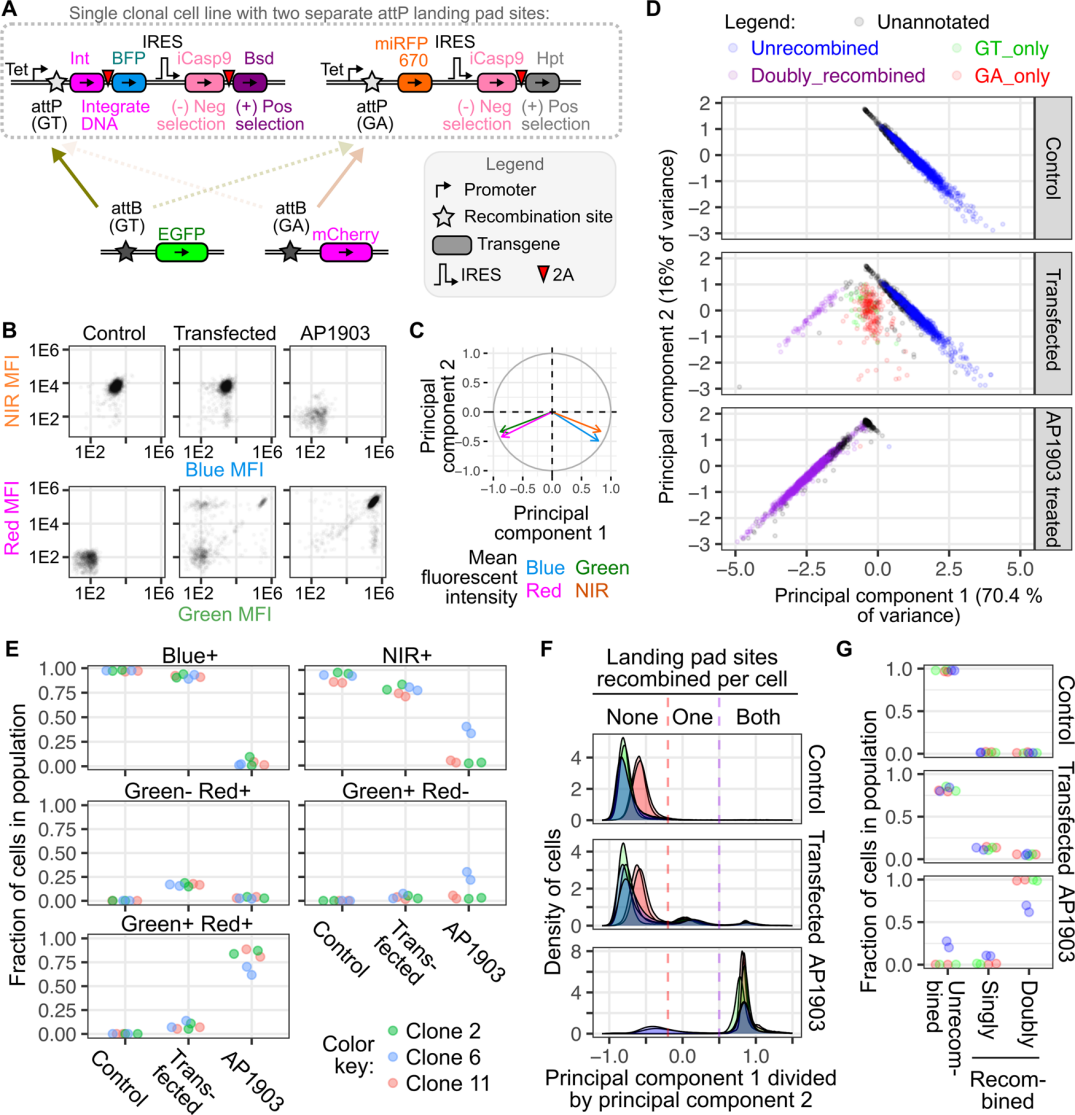
Dual landing pad cells for simultaneous single-copy transgene expression. (A) Schematic of the double landing pad cells. (B) Example flow cytometry scatter plots comparing near-infrared with blue fluorescence, or red with green fluorescence, for the double landing pad clone 11 cells prior to transfection (left), after transfection (middle), or after selection with AP1903 (right). (C) Weights of blue, green, red, and near-infrared intensity parameters within the first two principal components upon principal component analysis performed on flow cytometry data shown in panel B. (D) Location of individual cells from panel B shown as a scatter plot of the first two principal components. (E) Fractions of various fluorescent cell populations, prior to transfection, after transfection, or after selection with AP1903, for three different clones of double landing pad single cell clones, each independently transfected on two separate occasions. (F) Density plots of the ratiometric values of the first principal component of the data shown in panel E, divided by its second principal component. Threshold ratiometric values used to separate unrecombined, singly recombined, and doubly recombined cells are shown as dotted lines. A constant value of 2 was subtracted from principal component 2 to rescale the resulting ratiometric values and improve separation between populations. (G) Fractions of the unrecombined, singly recombined, and doubly recombined cells across the duplicate experiments for the three clonal double landing pad lines. Abbreviations: Tet, tetracycline-inducible promoter; Int, Bxb1 integrase; BFP, blue fluorescent protein; iCasp9, AP1903-inducible caspase 9; Bsd, blasticidin S deaminase; miRFP670, monomeric infrared fluorescent protein 670 nm; Hpt, hygromycin phosphotransferase; EGFP, enhanced green fluorescent protein; and mCherry, red fluorescent protein.

We next explored the possibilities of having the GT and GA landing pads present in a single copy in the same cell. To do this, we took existing HEK 293T GT attP LLP-Int-BFP-iCasp9-Blast cells^16,17^ and transduced them with lenti-landing pad particles, where the Bxb1 Int, BFP, and blasticidin resistance gene coding regions were swapped with miRFP670 and hygromycin resistance (Figure 3A). Blue and far-red fluorescent double landing pad clones were grown from single cells and used in subsequent recombination experiments, first focusing on clone 11 (Figure 3B). Upon transfecting the cells with a mixture of attB[GT]-EGFP and attB[GA]-mCherry recombination plasmids, we observed three new major populations of cells: red-only cells recombined at the GA landing pad only, green-only cells recombined at the GT landing pad only, and green and red cells recombined at both landing pads (Figure 3B). Since both landing pads encode iCasp9, the cells become resistant to AP1903-induced apoptosis only if recombination occurs in both landing pads. Indeed, AP1903 treatment selectively enriched green and red double-positive cells (Figure 3B). Roughly 7% of recombined and AP1903-treated cells exhibited green-high but red-low fluorescence (Figure 3B, bottom right). These are likely due to a population of the starting double landing pad cells exhibiting transcriptional downregulation at the attP[GA] landing pad locus, as there was a similar fraction of starting cells that exhibited blue-high but near-infrared-low fluorescence (Figure 3B, top left). Regardless, the vast majority of cells exhibited the maximal values of green and red fluorescence, demonstrating that we could create near-homogeneous populations of singly modified cells purely with iCasp9-based negative selection.

To simultaneously consider all four fluorescent parameters, including blue and near-infrared fluorescence, we performed principal component analysis on the resulting flow cytometry files. Principal component 1 separated the cells based on pre- and post-recombination fluorescent colors, while the second principal component separated cells based on their fluorescence intensity (Figure 3C). Consistent with what was observed with the individual pairwise scatter plots for fluorescence colors, the unselected transfected cells yielded a small population of double-positive cells along with single-color intermediates. Addition of AP1903 eliminated all cells except for those that were only double-positive for red and green or cells that exhibited no fluorescence at all (Figure 3D). This approach yielded populations of cells stably modified with each of the two different plasmids.

To assess the reproducibility of this method, we expanded our analysis to cover three different landing pad clones, each recombined on two separate occasions (Figure 3E). As all three clones are derived from cells with an identical GT landing pad site, they behaved indistinguishably for their loss of blue cells after recombination and selection. In contrast, all three clones exhibited similar recombination frequencies at their GA landing pad sites, but clone 6 exhibited only partial selection with AP1903 (Figure 3E). In the absence of selection, we found that transfection yielded ∼10% singly recombined cells and 8% doubly recombined cells. When clone 6 was ignored, selection with AP1903 yielded ∼85% doubly recombined cells.

To simultaneously consider all four colors, we applied the same principal component weights calculated on the first data set (shown in Figure 3C), on the full replicate data set. To aid in identifying various populations like those observed in Figure 3D, we calculated a ratiometric value, wherein the value for principal component 1 was divided by the value for principal component 2. This metric separated the unrecombined, singly recombined, and doubly recombined data points with a single statistic, aiding their quantification (Figure 3F). Across the six replicates, we found that the transfected populations were on average ∼13% singly recombined and ∼5.4% doubly recombined (Figure 3G). When clone 6 was ignored, selection with AP1903 yielded ∼99% doubly recombined cells by this analysis method (Figure 3G).

**Figure 4:**
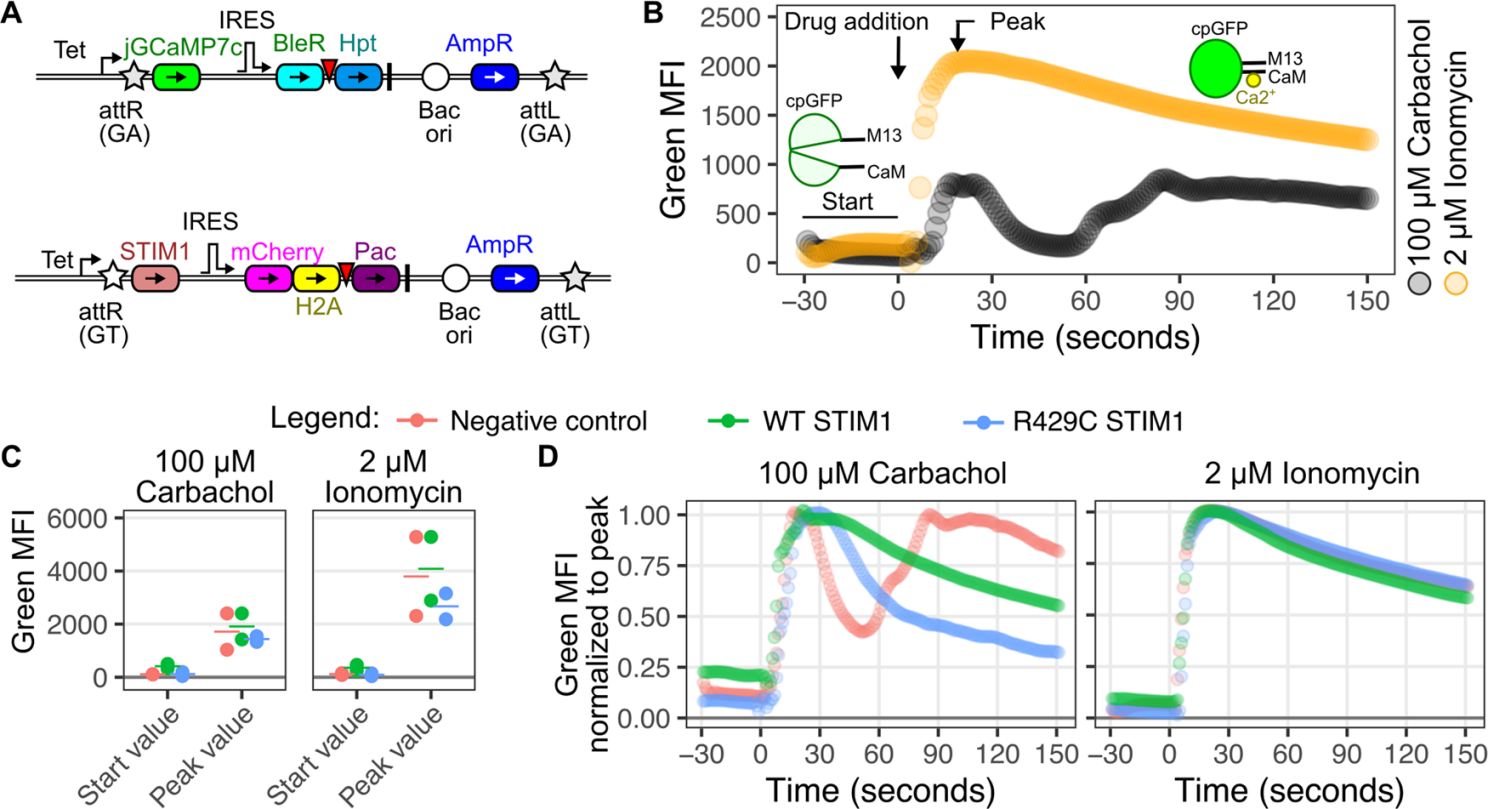
Assessing transgenes with protein biosensors using the dual landing pad. (A) Schematics of jGCaMP7c and STIM1 expression cassettes. (B) Representative plot of time-dependent jGCaMP7c fluorescence measurements, demonstrating changes upon the addition of Carbachol (black) or Ionomycin (orange). One replicate experiment with cells expressing a negative control cDNA construct is shown. (C) Plots showing individual replicate and mean values for the starting phases prior to drug addition, and peak values following drug addition, for cells overexpressing the negative control cDNA (red), WT STIM1 (green), or the loss-of-function STIM1 variant R429C (blue). (D) Plots of time-dependent jGCaMP7c fluorescence normalized to peak to demonstrate changes in fluorescence signal decay for negative control cDNA (red), WT STIM1 (green), or the loss-of-function STIM1 variant R429C (blue) after drug addition. All experiments were performed after first selecting for doubly recombined cells using AP1903, zeocin, and puromycin. Abbreviations: Tet, tetracycline-inducible promoter; jGCaMP7c, Janelia genetically encoded calcium indicator version 7c; IRES, internal ribosome entry site; BleR, bleomycin/zeocin resistance gene; Hpt, hygromycin phosphotransferase; Bac ori, bacterial origin of replication; AmpR, ampicillin resistance gene; STIM1, stromal interaction molecule 1 gene; mCherry, red fluorescent protein; H2A, histone 2A; Pac, puromycin *N*-acetyltransferase; cpGFP, circularly permuted green fluorescent protein; M13, peptide from myosin light-chain kinase; and CaM, calmodulin.

To demonstrate the utility of the double landing pad cells, we developed various multicomponent assays simultaneously requiring two transgenic cassettes. We first focused on engineering the double landing pad cells to express a transgenic cDNA of interest while simultaneously expressing a fluorescent biosensor that reports on the activity of the protein expressed from that cDNA. GCaMPs are genetically encoded calcium indicators (GECIs) that are widely used to measure calcium flux within the cell.^18^ They consist of circularly permuted GFP (cpGFP) flanked by the M13 fragment of myosin light-chain kinase at the N terminus and calmodulin on the C-terminus. The M13 fragment interacts with calcium-bound calmodulin, resulting in the conformational change and increased fluorescence intensity of cpGFP.^19^ We inserted jGCaMP7c^20^ into the GA landing pad (Figure 4A).

We focused on store-operated calcium entry (SOCE) as a source of calcium influx that can be measured with the genomically integrated jGCaMP7c. SOCE is typically induced through surface receptor activation and the subsequent depletion of calcium from the endoplasmic reticulum (ER). STIM1 is an ER-resident transmembrane protein that senses the loss of ER calcium stores and activates ORAI channels within the plasma membrane to permit a massive influx of calcium from the extracellular environment.^21^ We used carbachol, which mimics acetylcholine, to activate nicotinic and muscarinic receptors in our engineered cells and induce SOCE.^22^ Addition of carbachol to cells expressing jGCaMP7c from the GA landing pad site and a transgene unrelated to calcium biology in the GT landing pad site resulted in a biphasic-time-dependent curve of green fluorescence over time, measured by fluorescence microscopy. This curve is distinct from the monophasic curve after addition of ionomycin, a calcium ionophore used to immediately transport and increase calcium levels within the cell (Figure 4B).

Overexpression of STIM1 or its variants can perturb the SOCE-induced calcium influx. This includes the R429C variant of STIM1, which is unable to couple with ORAI and stimulate calcium influx itself. Furthermore, STIM1 R429C exhibits a dominant negative effect on endogenous STIM1,^23^ and the corresponding reduction in calcium influx in heterozygous individuals is associated with muscular hypotonia.^23^ To test the differences in calcium flux between STIM1 variants, we expressed either WT STIM1 or R429C STIM1 in the GT landing pad (Figure 4A) and measured the changes to jGCaMP7c fluorescence intensity following the addition of carbachol or ionomycin with fluorescence microscopy (Supporting Information Figure S3). The initial peak values of green fluorescence for WT STIM1 and the R429C variant-expressing cells were similar to the peak value of the negative control sample after the addition of carbachol, suggesting that the initial cytosolic influx of calcium was similar across conditions (Figure 4C). However, the time-dependent resolution of cytosolic calcium following this peak differed between samples. Unlike the biphasic curve for the negative control samples, which exhibited an initial peak around 20 s, rapid decline, and a second peak around 85 s, the STIM1 overexpressing cells were monophasic without the second peak (Figure 4D, left). The kinetics of green fluorescence decay for STIM1 overexpressing cells stimulated with carbachol was similar to cells treated with ionomycin (Figure 4D, right). In contrast, cells overexpressing the R429C STIM1 variant exhibited a much faster decay in fluorescence with carbachol, likely through its inability to maintain the opening of the ORAI channels. Overall, these results show that the double landing pad cells can be used to pair genetically encoded biosensors with transgenic overexpression to perturb and study intracellular signaling pathways.

**Figure 5:**
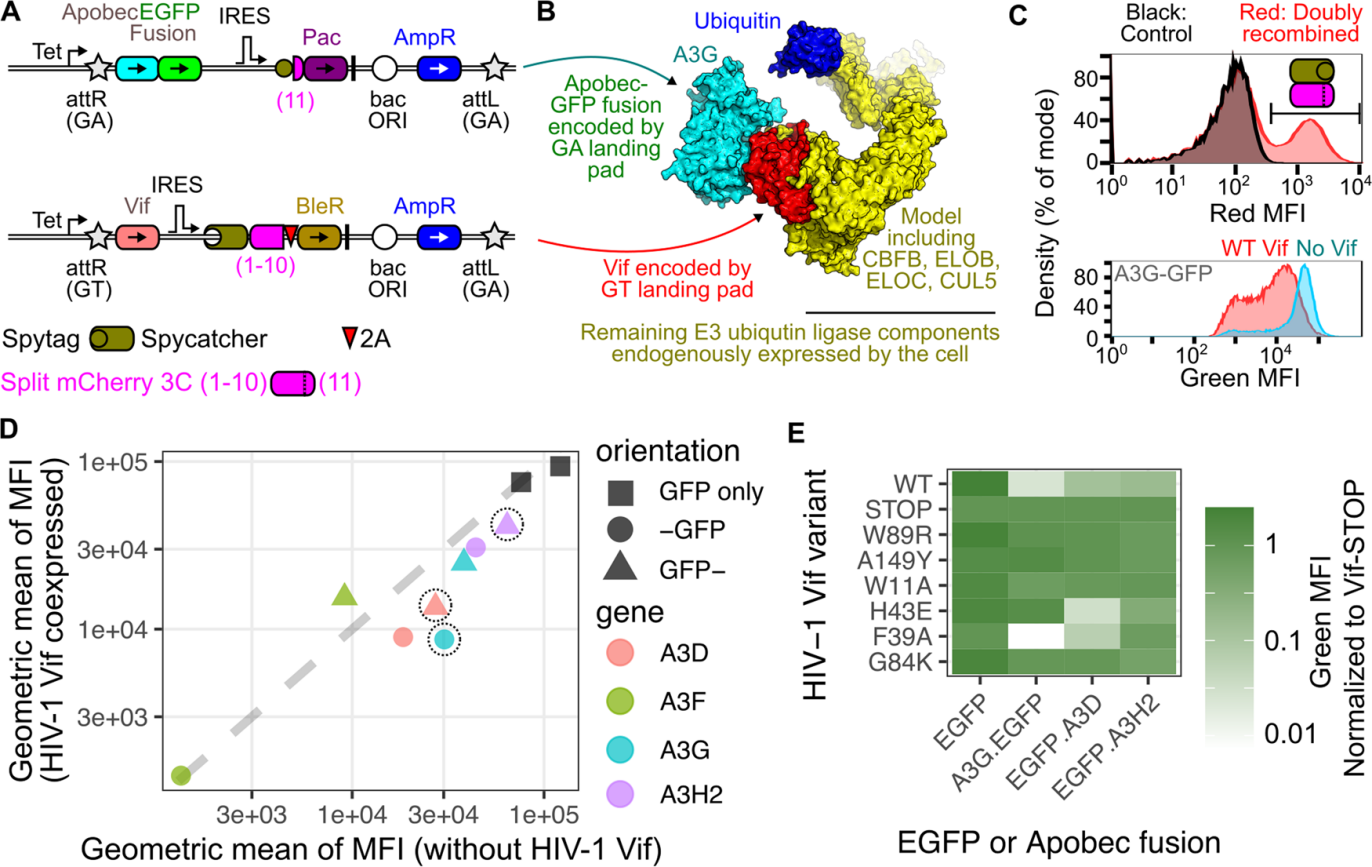
Studying pairs of interacting proteins with the dual landing pad. (A) Schematic of the GA and GT recombination sites, encoding either an EGFP-fused Apobec gene or HIV-1 Vif. (B) Schematic showing which protein products are created from the landing pads or endogenously expressed within the cells. The E3 ubiquitin ligase complex model was created through merging PDBs 8CX0, 4N9F, and 7B5M. (C) Top: Red fluorescence from cells expressing both Spytag-sfmCherry(11) and Spycatcher-sfmCherry3C(1–10), as compared to control cells lacking red fluorescence. Bottom: Doubly recombined red fluorescent cells recombined with A3G-EGFP and were further assessed for green fluorescence, with distributions shown for cells expressing a prematurely truncated Vif (No Vif; blue) or WT Vif (red). (D) Scatter plot showing the geometric means of each Apobec protein either N- or C-terminally fused to GFP, either in the presence (*Y*-axis) or absence (*X*-axis) of HIV-1 Vif. Symbols surrounded by dotted circles denote orientations that were used to test the larger panel of HIV-1 Vif mutants in subsequent experiments. Black points are GFP only without Apobec3 fusion tested separately two different times. Points below the dotted line denote situations where Vif expression reduced Apobec steady-state abundance. (E) Heat map showing the amounts of green fluorescence observed in each Vif and GFP-fused Apobec protein combination, normalized to cells encoding a nonfunctional Vif protein with a premature stop codon. The results are the geometric mean of three replicate experiments. All experiments were performed after selecting for doubly recombined cells using AP1903. Abbreviations: Tet, tetracycline-inducible promoter; Apobec, Apolipoprotein B mRNA Editing Catalytic Polypeptide-like family 3 gene; EGFP, enhanced green fluorescent protein; IRES, internal ribosome entry site; Pac, puromycin resistance gene; BleR, bleomycin/zeocin resistance gene; bac ORI, bacterial origin of replication; AmpR, ampicillin resistance gene; Vif, viral infectivity factor; sfmCherry3c(1–10), superfolder mCherry red fluorescent protein version 3c split to encode only β sheets 1 through 10; and sfmCherry(11), superfolder mCherry red fluorescent protein split to encode only β sheet 11.

We also focused on assays requiring the combinatorial testing of two different protein partners. The Apobec proteins are a family of cytosine deaminases, with the Apobec3 (A3) paralogs serving as innate immune proteins that protect cells against viruses like human immunodeficiency virus type 1 (HIV-1).^24−29^ At least four human A3 paralogs can exhibit anti-HIV activity: Apobec3G (A3G),^24−29^ Apobec3D (A3D),^30^ Apobec3F (A3F),^31^ and Apobec3H (Haplotype 2; A3H2).^32^ In response, HIV-1 has evolved viral infectivity factor (Vif), which targets the A3 proteins for proteasomal degradation by serving as a molecular glue tethering them to the Cullin5-ElonginB-ElonginC-Rbx2 E3 ubiquitin ligase complex for polyubiquitination (Figure 5B).^33,34^ Various Vif mutants show differential effects for the human A3 paralogs,^35−38^ demonstrating that the interacting sites between HIV-1 Vif and each A3 paralog are often overlapping but at least partially distinct. The A3 protein degradation observed when bound by Vif is traditionally assessed by Western blotting. A handful of studies have harnessed fluorescent protein fusions to the A3 proteins to increase experimental throughput and perform cell-based screens for small molecule inhibitors of this interaction.^39,40^ No studies have combined A3 fluorescent protein fusions with panels of Vif mutants to test the pairwise interactions between these proteins at high throughput.

To pilot this assay, we created a set of GA attB constructs systematically fusing EGFP to either the N- or C-terminus of A3G, A3D, A3F, or A3H2. We also made a complementary pair of GT attB constructs, encoding either WT Vif or a prematurely truncated, nonfunctional mutant (Figure 5A). Each GT attB and GA attB construct encoded half of a split mCherry protein fused to either Spytag or Spycatcher to enhance mCherry reconstitution, where only cells recombined with both constructs yielded red fluorescence (Figure 5C, top). We measured the levels of green fluorescence observed for each construct in the presence or absence of Vif (Figure 5C, bottom). As expected, cells encoding only EGFP in the absence of A3 fusion partners were equally fluorescent in the presence or absence of Vif (Figure 5D). A3F-GFP was undetectable, while GFP-A3F did not exhibit Vif-dependent degradation. A3G-GFP exhibited greater dynamic range than the opposite orientation. The orientation of GFP fusion did not exhibit a differential effect on A3D or A3H2 degradation, although the N-terminal GFP format gave an overall total signal (Figure 5D).

We tested the most informative A3 fusions with a panel of 8 total Vif sequence variants (Figure 5E and Supporting Information Figure S4). The W89R and A149Y mutants reduce association with the Cullin5 E3 ubiquitin ligase complex.^33^ As expected, these mutants lost activity against all three tested A3 paralogs. W11A also exhibited little to no activity against all three paralogs, despite being published to lose activity toward only A3F but not A3G.^41^ H43E selectively lost activity toward A3G, consistent with the known role of His43 in this interaction.^35^ F39A only lost activity against A3H2, consistent with the known role of Phe39 in this interaction.^37,38^ G84K lost all activity against A3G and A3D and retained only minor activity against A3H2. This was consistent with previous work demonstrating that Gly84 is involved in binding both A3G and A3D.^42^ Overall, our proof-of-principle experiments harnessing the double landing pad cells largely recapitulated published effects of Vif coding variants on human A3 paralog degradation, demonstrating the applicability of this approach to comprehensively characterize the effects of HIV-1 Vif coding mutants on A3 protein interactions and, by extension, host cell antiviral innate immunity.

Finally, to enable future expansion into even more complicated expression systems, we assessed the orthogonality of additional central dinucleotide pairs for their orthogonality. Various in vitro studies with purified Bxb1 enzyme and oligonucleotides encoding recombination sites with different central dinucleotides demonstrated that each dinucleotide exhibits slightly different mechanistic characteristics.^43,44^ We thus created landing pad plasmids for each of the possible GN dinucleotide attP sites and cotransfected each plasmid with an equimolar mixture of recombination plasmids encoding each of the central GN dinucleotide attB sites. We harvested DNA from the cells and sequenced the resulting attR junctions to calculate the relative rates of recombination for each attB plasmid for each attP site.

**Figure 6:**
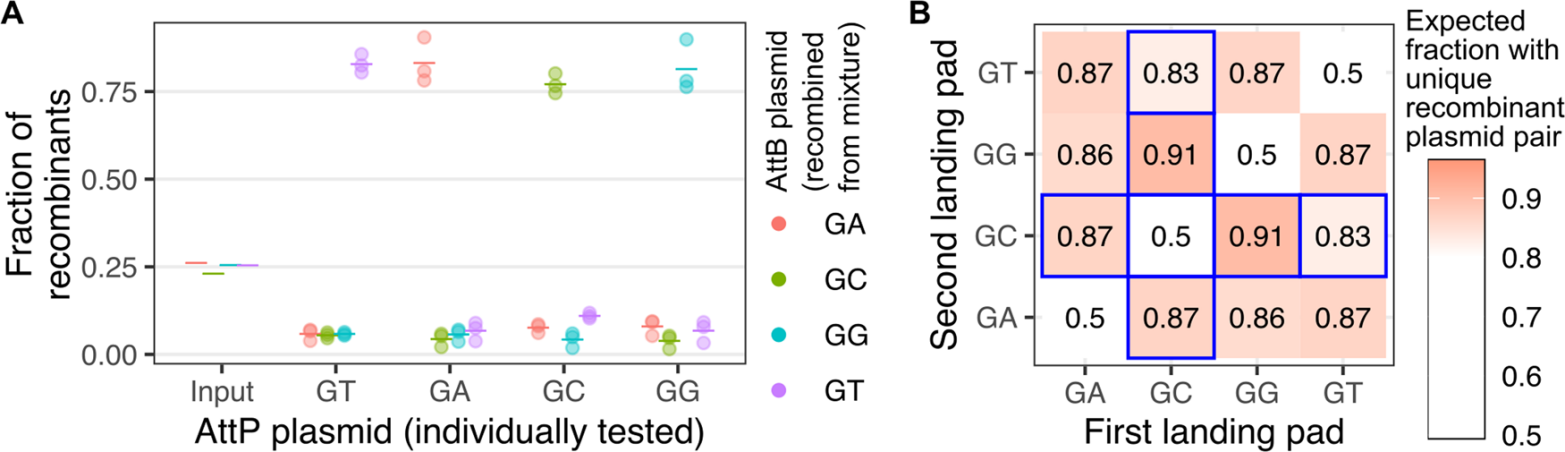
Additional orthologous Bxb1 central dinucleotide pairs for future analysis. (A) Fraction of Illumina sequencing reads corresponding to recombination with the indicated dinucleotide attB recombination plasmids. Different attP plasmids that were individually tested are labeled along the *X*-axis, while the identity of each recombination plasmid within the mixture is shown as different colors. Each colored horizontal line is the geometric mean of 3 replicate experiments, while the colored points show individual replicate values. The horizontal lines above “input” are the results of sequencing the plasmid mixture prior to transfection, confirming that the attB-harboring plasmids were mixed in a roughly equimolar ratio. This experiment was performed in the absence of any selection steps. (B) Hypothetical fraction of uniquely recombined double landing pad cells expected based on the plasmid recombination rates shown in panel A. This was achieved by simulating two independent sampling events based on the geometric mean recombination rates shown in panel A, repeating this process 100,000 times, and calculating the total number of times the correct pair of sequences was found together. The blue outline demarcates combinations involving the GC central dinucleotide, as the palindromic nature of this dinucleotide affects the rate of correct directionality of resulting transgenic integration.

The GA and GT dinucleotide sites exhibited the greatest selectivity at being correctly paired 83% of the time (Figure 6A). The GG dinucleotide performed slightly less stringently at 81% selectivity, while the GC dinucleotide was the most promiscuous at 77% selectivity (Figure 6A). The palindromic GC dinucleotide generates equal amounts of correct attL and attR sites and aberrant BP and B′P′ products in vitro, wherein the left half of attP is joined to the left half of attB (BP) and the right halves of attP and attB are joined (B′P′),^44^ and the direction of the transgenic cargo is inverted. Due to this inversion, the aberrant BP and B′P′ products were not captured in our amplification and sequencing design and may explain the seemingly higher proportions of mismatched attR and attL sites observed with this attP sequence.

When simultaneously using multiple landing pads with differing dinucleotide sites, such as with our double landing pad cells, one can use these rates of on- and off-target recombination to predict which Bxb1 central dinucleotide pairs would generate the greatest number of cells integrated with the desired pair of unique plasmids rather than two copies of the same plasmid through mismatched recombination. Using the aforementioned rates of matched and mismatched recombinations, we simulated the relative fractions of correctly recombined plasmids expected with any given pair of double landing pads. If avoiding the GC dinucleotide site, the remaining pairwise combinations of GT, GA, or GG all had similar accuracy, where a unique pair of plasmids were integrated into the cell roughly 85 or 86% of the time (Figure 6B). Notably, the simulated values for the on- and off-target recombinations for the GA and GT pair were lower than our empirically determined values, which were ∼99% (Figure 1B). Thus, it is likely that all pairwise combinations of GT, GA, or GG landing pads would exhibit similar on-target rates. Overall, the orthogonality and consistent directionality of the GT, GA, and GG sites suggest potential routes for generating future landing pad cells with even more complex capabilities for genetic modification.

## Discussion

“Landing pad” approaches harnessing site-specific DNA recombinases provide a convenient and reproducible method for the stable genetic engineering of cultured mammalian cell lines. The characterization of multiple orthogonal recombination sites has enabled more complex engineering strategies, such as recombinase-mediated cassette exchange (RMCE)^15,45,46^ or the independent integrations of more than one transgenic payload.^47^ A wealth of existing recombinase literature has demonstrated both approaches for tyrosine recombinases such as Cre and Flp,^2,46,48−51^ with more recent studies now incorporating Bxb1.^15,47,52^ Here, we further explored the engineering strategies possible with a single Bxb1 recombinase enzyme when combined with orthogonal pairs of recombinase sites.

Traditional RMCE strategies use a pair of orthogonal recombination sites both in the genomic landing pad and flanking a plasmid-based transgenic cassette of interest. We took a conceptually similar but practically distinct approach, wherein genomic integration of a plasmid molecule occurs through a single attP-attB pair, but the undesired bacterial sequence within the plasmid is flanked by an orthogonal attP-attB pair and is thus excised from the final engineered product. This approach was efficient, as more than 95% of genomically integrated plasmids had excised undesirable bacterial sequences. While similar in the final outcome as traditional RMCE approaches, the flanked plasmid approach can instead be performed with existing cells harboring a single recombinase site, where only the plasmid needs to be specifically engineered when removal of bacterial sequence is paramount to the experiment.

These experiments also yielded unexpected insights into the transcriptional readthrough. When using AP1903 paired with iCasp9 to remove unrecombined cells, we found that cells integrated with entire, unexcised plasmids survived better than those with the bacterial portion removed (Supporting Information Figure S2). This was likely due to an additional SV40 terminator element that, when left unexcised within the plasmid DNA, likely combined with the downstream TK terminator element to prevent transcriptional readthrough into the downstream IRES-iCasp9 cassette (Supporting Information Figure S2). This observation suggests that it may be prudent to utilize multiple transcriptional terminators when an engineering strategy dependent on iCasp9 downregulation is used through distancing from a strong transcriptional promoter.

While “double landing pad cells” have been described before, these methods have either used tyrosine recombinases such as Cre or Flp with well-characterized heterospecific sites^50^ or multiple recombinases with their own unique recombination sites,^15,47^ to achieve multicassette insertion. By using a single, highly efficient Bxb1 recombinase with more than one orthogonal recombinase site, this simplifies the steps needed to integrate into both landing pad sites while also benefiting from the highest possible recombination efficiencies. Furthermore, unlike cells generated for the high-yield production of biological molecules, the cells we created were engineered for multiplexable functional assays, where it is critical that only a single copy of each landing pad be integrated into each cell line. Our strategy of low multiplicity of infection landing pad lentiviral transduction paired with downstream fluorescent protein or antibiotic resistance logic gates all but ensures the generation of precisely modified cells.

We demonstrated the utility of double landing pad cells with two different use cases. We showed how the second landing pad site could be used to express a cellular biosensor, such as the calcium-sensing jGCaMP7c fluorescent protein.^20^ By having the biosensor element easily interchangeable, it is easy to update the experimental format as improved biosensors are discovered, such as the new calcium indicator NEMOc.^53^

This assay format could be paired with a site-saturation mutagenesis library of a protein capable of modulating intracellular calcium concentrations such as STIM1. Similar biosensor–modulator pairs could easily be tested in the future, such as cyclic adenosine monophosphate (cAMP) indicators^54^ to study variants of GNAS^55^ in pseudohypoparathyroidism, or pyruvate sensors^56^ to study variants in PDHA1 involved in pyruvate dehydrogenase complex deficiency.^57^

We also demonstrated that the two landing pads could be used to express the protein interaction parts. This is particularly helpful when there are multiple versions of each protein partner, so that the full range of complexity can be assessed by testing combinations of two smaller libraries rather than being forced into encoding each possible combination on the same plasmid molecule through more laborious molecular cloning techniques. We suspect that this technique will be particularly helpful for studying complex dimeric interactions. For example, the double landing pad configuration can be used to study integrin binding, where one landing pad could be used to express one of 8 possible integrin β subunits, while the other can be used to express one of the 18 different integrin α subunits. While there are only 24 integrins known to be made in humans,^58^ this format would allow the testing of all 144 possible combinations to exhaustively characterize how various integrin heterodimers bind the full range of known or suspected ligands.

There will likely be future innovations that are possible with incorporating additional orthogonal recombination sites. We only assessed the orthogonality of the four Bxb1 central dinucleotides starting with G, since the frequencies of matched and mismatched recombination sites could be calculated by sequencing a single attL or attR sequence, but in vitro studies have shown that both nucleotides could be varied, yielding 16 possible combinations with Bxb1 alone. Avoiding palindromic dinucleotides such as GC, which yielded a mixture of integrated DNA in the forward and reverse orientations,^44^ still leaves 12 nonpalindromic dinucleotide combinations that can be used. Assessing the all-by-all orthogonality of these sites would require simultaneous sequencing of both attL and attR sequences within the same cell, which is difficult with current short-read DNA sequencing technologies. This flexibility will likely become more important as various scientific applications mature and require more complicated transgene expression schemes.

## Methods

### Recombinant DNA Construction

All plasmids were produced via Gibson Assembly.^59^ Construction of our landing pad lentiviral vector construct, LLP-Int-BFP-IRES-iCasp9-Blast (Addgene plasmid #171588), was described previously.^16^ For initial amplification, a total of 40 ng of plasmid DNA was mixed with 0.333 μM of each forward and reverse primer, and 2× Kapa HiFi HotStart ReadyMix (Roche, KK2602) was added as half the volume. DNA amplification occurred under the following conditions: 95 °C 5′, 98 °C 20″, 65 °C 15″, 72 °C 8′, repeat seven or eight times, 72 °C 5′, 4 °C hold. A total of 1 μL (20 units) of DPNI enzyme (New England Biolabs, R0176L) was added to each sample and incubated at 37 °C for 2 h. The reactions were cleaned with a Zymo clean and concentrator kit (Zymo Research, D4003). For two- and three-part Gibson reactions, the amplicons were purified together in the same column, and then 1 μL of each eluate was mixed together with 1 μL of 2× GeneArt Gibson Assembly Master Mix (Thermo Fisher, A46629) and incubated at 50 °C for 60 min. The recombinant plasmids were transformed into homemade chemically competent *Escherichia coli* 10-β (New England BioLabs, C3019I). Transformation efficiency was calculated as the number of colony forming units per nanogram of plasmid determined by dilution plating and colony count. Plasmids were extracted with a GeneJet miniprep kit (Thermo Fisher, K0503) and sequence-confirmed via Sanger sequencing on an Applied Biosystems 3730 genetic analyzer, followed by whole-plasmid sequencing by Plasmidsaurus via long-read sequencing from Oxford Nanopore Technologies. All landing pad constructs used in this work encode a CMV-rtTA3G cassette driving the Tet inducible promoter, as shown in Figure 1A.

The following plasmids were procured from Addgene and used as template DNA molecules to create the plasmid constructs used in this work: 1066 pBabe puroL PTEN was a gift from William Sellers (Addgene plasmid # 10785; http://n2t.net/addgene:10785; RRID:Addgene_10785); pCDNA3_UnaG-Flag-Sec61B was a gift from Hyun-Woo Rhee (Addgene plasmid # 83413; http://n2t.net/addgene:83413; RRID:Addgene_83413);^60^ pLEX-MCS-ASC-GFP was a gift from Christian Stehlik (Addgene plasmid # 73957; http://n2t.net/addgene:73957; RRID:Addgene_73957);^61^ pcDNA3.1(+)_SpyCatcher-6aa-sfCherry2(1–10) was a gift from Bo Huang (Addgene plasmid # 117484; http://n2t.net/addgene:117484; RRID:Addgene_117484);^62^ gRNA_Cloning Vector was a gift from George Church (Addgene plasmid # 41824; http://n2t.net/addgene:41824; RRID:Addgene_41824);^63^ pSFFV_sfCherry3C(1–10) was a gift from Bo Huang (Addgene plasmid # 117482; http://n2t.net/addgene:117482; RRID:Addgene_117482);^62^ pGP-CMV-jGCaMP7c variant 1513 was a gift from Douglas Kim & GENIE Project (Addgene plasmid # 105320; http://n2t.net/addgene:105320; RRID:Addgene_105320);^20^ and pLNCX2-STIM1 was a gift from Shengyu Yang (Addgene plasmid # 89817; http://n2t.net/addgene:89817; RRID:Addgene_89817).^64^

The following plasmids were created as part of this work and deposited into Addgene: G384A_pLenti-Tet-BattP(GT)-BFP-2A-iCasp9-2A-Blast_rtTA3 (Addgene plasmid #200630); G417A_pLenti-Tet-BattP(GA)-BFP-2A-iCasp9-2A-Blast_rtTA3 (Addgene plasmid #200631); G413A_BattB(GA)-EGFP (Addgene plasmid #200632); G421C_BattB(GA)_mCherry (Addgene plasmid #200633); G718A_AttB(GA)_TKterm_AttB(GT)_sGFP-PTEN-IRES-mCherry-P2A-HygroR_noTerm_attP(GA)_ IRES-UnaG-2A-Puro-Term (Addgene plasmid #200634); G783A_pLenti-Tet-BattP(GA)-miRFP670-2A-iCasp9-2A-Blast_rtTA3 (Addgene plasmid #200635); G1052C_AttB(GA)_EGFP_IRES-Spycatcher- sfmCherry3C(1–10)-P2A-shBleR (Addgene plasmid #200636); J135F_AttB_Vif(T27 × 4)-IRES-SpyTag- sfmCherry2(11)-PuroR (Addgene plasmid # 200637); G1095C_AttB(GA)_jGCaMP7c_IRES_shBleR-P2A- HygroR (Addgene plasmid #200638); and pNK005C_AttB_mkozak_STIM1_IRES_mCherry-H2A-P2A-PuroR (Addgene plasmid #200639).

### Cell Culture and Landing Pad Clone Generation

All cell culture reagents were purchased from Thermo Fisher unless otherwise noted. The medium used for culturing all cell lines, denoted as “D10”, consisted of Dulbecco’s modified Eagle’s medium supplemented with 10% fetal bovine serum (Gibco, 10437028), 100 U/mL penicillin, and 0.1 mg/mL streptomycin (Corning, 30-002-CI). Cells were passaged by detachment with trypsin-ethylenediaminetetraacetic acid 0.25% (Corning, 25-053-CI).

Generation of a HEK 293T-based cell line containing a single lenti-landing pad, LLP-Int-BFP-IRES-iCasp9-Blast, was previously described.^16^ For the dual landing pad system, we developed a clonal cell line from the existing single landing pad cell line so that each cell would contain a single copy of both the GT landing pad and the GA landing pad. To do this, single landing pad cells were transduced with lentivector supernatants containing LLP-attP(GA)-miRFP670-2A-iCasp9-2A-Hpt at various dilutions, ranging from 30 μL to 3 mL. One day post-transduction, the media was switched to D10 containing 2 μg/mL doxycycline (D10-dox). At least 3 days after transduction, the cells were visually confirmed for BFP expression with fluorescence microscopy, and flow cytometry was used to verify miRFP670 expression, as well as confirm that the cells were transduced at a multiplicity of infection clearly less than one. The cells were then selected with a mixture of 10 μg/mL blasticidin and 100 μg/mL hygromycin, and it was visually confirmed that the majority of cells died off, further supporting the culture to have been transduced at a multiplicity of infection less than one. Colonies of clonal cells were picked from the plate and allowed to expand in their own well. To verify dual integration, we recombined the cells with a mixture of attB[GT]-EGFP and attB[GA]-mCherry recombination plasmids, selected for recombinants, and identified a clone with dual integration into both landing pads via flow cytometry.

### Recombination of Landing Pad Cells

Depending on the experiment, single or dual landing pad cells were used for Bxb1-mediated recombination via plasmid transfection using the Xfect transfection reagent from Takara Bio (catalog # 631318). The day before transfection, approximately 180,000 cells were seeded into a 24-well plate with a total of 300 μL of D10-dox media. For transfection of attB plasmids, 1 μg of DNA was diluted in Xfect reaction buffer to a total volume of 25 μL with 0.3 μL of Xfect polymer. The mixture was incubated in the cells at 37 °C for at least 4 h or overnight before being removed or diluted out.

Following attB plasmid transfection, the negative selection of unmodified landing pad cells was performed with the addition of 10 nM AP1903 (ApexBio, B4168) to activate iCasp9. Positive selection of recombined cells was achieved with the addition of 0.75 μg/mL puromycin (InvivoGen, ANTPR1) and/or 50 μg/mL Zeocin (Fisher Scientific, R25001).

### Flow Cytometry

Cells were detached with 0.25% Trypsin with 2.21 mM EDTA (Corning, #25-053-CI) and resuspended in PBS containing 5% fetal bovine serum. Cytometry was performed with a Thermo Fisher Attune NxT flow cytometer. During data acquisition, mTagBFP2 was excited with a 405 nm laser, and emitted light was collected after passing through a 440/50 nm bandpass filter. EGFP was excited with a 488 nm laser, and emitted light was collected after passing through a 530/30 nm bandpass filter. mCherry was excited with a 561 nm laser, and emitted light was collected after passing through a 620/15 nm bandpass filter. iRFP670 and miRFP670 were excited with a 638 nm laser, and emitted light was collected after passing through a 720/30 nm bandpass filter. Before analysis of fluorescence, live, single cells were gated using FSC-A and SSC-A (for live cells) and FSC-A and FSC-H (for single cells). In most cases, recombined cells were subsequently gated by identifying populations of green or red cells. For the Vif-Apobec samples, the cells were first gated for a lack of blue or near-infrared fluorescence prior to gating for red fluorescence. They were then gated for green positivity prior to calculating the geometric mean MFI for that sample to control for the variable amounts of residual silenced cells that may have made it through the gating scheme and could skew the results unless removed.

### Fluorescence Microscopy

Fluorescent images were captured on a Nikon Ti-2E fluorescent microscope, outfitted with a SOLA SM II 365 light engine (Lumencor), a CFI Plan Apochromat DM Lambda 20X objective or a NIKON Plan Fluor 4× objective, and GFP (No. 96392), Texas Red (No. 96395), or Cy5 (No. 96396) filter sets, and imaged with a DS-QI2 monochrome CMOS camera. Double landing pad cells were recombined with jGCaMP7c in the GA site and either a plasmid encoding mCherry by itself or mCherry along with STIM1 WT or STIM1 R429C in the GT site. Cells were grown in D10 media containing 1.8 mM calcium and 0.8 mM magnesium. Cells were plated in a 96-well plate in D10 media for image capture. Images were captured at 1 s per image for 3 min with an exposure time of 100 ms using NIS-Elements AR version 5.21.01. 100 μM carbachol (Millipore Sigma, PHR1511) or 2 μM ionomycin (Alfa Aesar, J62448) final concentration was added to cells at 30 s after the start of acquisition. NIS-Elements AR software was used for the analysis of the time series images. Regions of interest were drawn around five individual cells and the background for each condition in each replicate experiment. Signal values were calculated by generating mean intensity time series graphs for each cell, and values at each second were averaged and subtracted by the background values. The resulting time-dependent mean values were imported into R for subsequent analysis.

### High Throughput DNA Sequencing

Once selection for singly or doubly recombined cells was complete, cells were harvested and genomic DNA was extracted using a Qiagen DNeasy Blood and Tissue Kit (catalog # 69506). The DNA segments of interest were amplified from 500 ng of DNA with 0.3125 μM forward and reverse primer in a 40 μL reaction with Phusion Flash high-fidelity PCR Master Mix (Thermo Scientific, F548L) added as half the volume. Reaction conditions were 95 °C 3′, 95 °C 15″, 60 °C 15″, 72 °C 30″, repeated 26 to 28 times, 72 °C 1′, 4 °C hold. In the case of excision via flanking GA sites, an extra forward or reverse primer was used so that both excised and unexcised products could be amplified from the same PCR tube.

For these reactions, the extension time was reduced to 10 s. A table of the primer names and sequences used for amplification can be found in Supporting Information Table 1. PCR products were obtained on a 2% TAE-agarose gel. DNA bands were excised and extracted using a GeneJet Gel Extraction and DNA Cleanup Micro Kit (Thermo Scientific, K0832). A Qubit dsDNA BR Assay Kit (Invitrogen, Q32850) was used to quantify the DNA in the extracted bands on a Qubit 4 fluorometer (Invitrogen, Q33238). The DNA regions of interest were sequenced by the Amplicon-EZ service provided by Genewiz (now Azenta Life Sciences), providing Illumina high throughput sequencing with read 1 and read 2 sequences of 250 nt each.

The read 1 and read 2 fastq files from each sample were paired using PEAR v0.9.6.^65^ The relative frequencies of the formation of various recombined DNA products were assessed by counting the frequency of particular DNA sequences in the resulting paired fastq files by using the grep function in Bash. The fastq reads have been deposited into NCBI GEO under accession number GSE231138.

For the excision experiments using primer set 1, excised plasmids were identified by searching for nucleotides on the right side of the attB(GA) site “ACTCCGTCGTCAGGATCATCC,” while unexcised plasmids were identified by searching for nucleotides on the right side of the attP(GA) site “ACTCAGTGGTGTACGGTACAAACC.” For the excision experiments using primer set 2, excised plasmids were identified by searching for nucleotides immediately to the left of the attP(GA) site “GATCCCCTTTAGTGAGGGTT,” while unexcised plasmids were identified by searching for nucleotides on the left of the attB(GA) site “CGACGATGTAGGTCACGGCA.”

For the orthogonal GN dinucleotide experiments, the relative numbers of recombination events for the attB sequences of each central dinucleotide within the mixture were determined by searching for and counting the instances of each resulting attR sequence present in the read. For replicates 2 and 3, each sample containing a different central dinucleotide attP sequence was amplified with a unique primer marking that sample. For example, samples where the attP sequence has a central GG dinucleotide were amplified with a primer, adding a G nucleotide as the first nucleotide of read 1. This allowed us to further multiplex samples, reducing the overall sequencing cost.

### Data Analysis and Statistics

Data analysis was performed using version 2023.03.0 + 386 of RStudio running version 4.2.2 of R, with the exception of flow cytometry data, which was first analyzed using version 10.8.1 of FlowJo. An R Markdown file containing code capable of fully reproducing the analyses can be found at the Matreyek Lab GitHub repository (https://github.com/MatreyekLab/Recombinastics). The analysis utilized the tidyverse,^66^ ggrepel,^67^ ggbeeswarm,^68^ abind,^69^ gridExtra,^70^ patchwork, and reshape^71^ packages.

### Simulation of Pairwise Combinations of Double Landing Pad Recombinations

The simulation was performed in R and estimates the relative fraction of cells expected to produce the intended recombination outcome, where one each of two plasmid mixtures is integrated into one site of cells harboring two landing pad loci. We systematically examined each pairwise combination of GN central dinucleotide recombinase sites. In cases where the same central dinucleotide was being considered for both landing pads, a value of 0.5 was assigned, as the probability of having one of each plasmid is equivalent to the probability that a cell has two copies of one plasmid and zero copies of the other. In cases where different central dinucleotides combinations were being considered, the on- and off-target recombination frequencies for the two GN sites in question were taken from the values determined in the GN 4-plasmid mixture recombination experiments shown in Figure 6A, and used as probabilities to simulate 100,000 different recombination events for that pair. The number of simulation events that yielded unique plasmids integrated at each site was summed and divided by the total number of simulations to yield the estimated fraction of intended integration.

## Supporting Information Available

Detailed schematic of pared or integrated products possible with the GA attB flanked construct; results from integration of the GA site flanked construct in LLP-Int-iCasp9-Blast cells; fluorescence intensities of carbachol and ionomycin treated cells without normalization; and replicate normalized GFP intensity values (PDF)
Primer names and sequences used for amplification prior to high throughput Illumina sequencing (XLSX)

## Abbreviations

RMCE: recombinase-mediated cassette exchange
MAVE: multiplex assays of variant effect
DMS: deep mutational scanning
A3G: Apobec 3G
A3F: Apobec 3F
A3D: Apobec 3D
A3H2: Apobec3H haplotype 2
Tet: tetracycline-inducible promoter
BFP: blue fluorescent protein
iCasp9: AP1903-inducible caspase 9
Bsd: blasticidin S deaminase
CMV: cytomegalovirus immediate early promoter
rtTA3G: reverse tet transactivator third generation
PTEN: phosphatase and tensin homologue gene as transgenic cargo
mCherry: red fluorescent protein
IRES: internal ribosome entry site
Hpt: hygromycin phosphotransferase gene
UnaG: green fluorescent protein derived from eel
2A, P2A: viral translational stop-start sequence
Pac: puromycin *N*-acetyltransferase
Bac ori: bacterial origin of replication
AmpR: ampicillin resistance gene
SV40 term: transcriptional terminator from simian virus 40
TKterm: transcriptional terminator from herpes simplex virus thymidine kinase
Int: Bxb1 integrase
miRFP670: monomeric infrared fluorescent protein 670 nm
EGFP: enhanced green fluorescent protein
jGCaMP7c: Janelia genetically encoded calcium indicator version 7c
BleR: bleomycin/zeocin resistance gene
STIM1: stromal interaction molecule 1 gene
H2A: histone 2A
cpGFP: circularly permuted green fluorescent protein
M13: peptide from myosin light-chain kinase
CaM: calmodulin
Apobec: apolipoprotein B mRNA editing catalytic polypeptide-like family 3 gene
Vif: viral infectivity factor
sfmCherry3c(1–10): superfolder mCherry red fluorescent protein version 3c split to encode only β sheets 1 through 10
sfmCherry(11): superfolder mCherry red fluorescent protein split to encode only β sheet 11
