# Supplementary material for: Mammalian Genomic Manipulation with Orthogonal Bxb1 DNA Recombinase Sites for the Functional Characterization of Protein Variants
Source: ACS Synth Biol. 2023 Nov 3;12(11):3352–65. doi: 10.1021/acssynbio.3c00355 (PMC10661055; doi:10.1021/acssynbio.3c00355)
Supplement: Supplementary file 1 — sb3c00355_si_001.pdf [file sb3c00355_si_001.pdf]

Supporting Information

Mammalian genomic manipulation with orthogonal Bxb1 DNA recombinase sites for the functional characterization of protein variants

Sarah M. Roelle[a], Nisha D. Kamath[a], Kenneth A. Matreyek[a*]

[a] Department of Pathology, Case Western Reserve University School of Medicine, Cleveland, Ohio, 44106, USA

[*] Email: kenneth.matreyek@case.edu

Supplementary Table 1. Primer names and sequences for DNA amplification prior to high throughput sequencing. The Supplementary Table is supplied in a separate .xlsx excel file. Column headers: “Type” refers to the application the primer was used for; “Direction” refers to whether it was the “forward” or “reverse” primer within the primer set for that reaction; “Adapter” sequence refers to the Amplicon-EZ adapter sequence appended to the hybridization sequence for the primer; “Construct” refers to the plasmid or, in the case of genomically integrated DNA, the reconstructed DNA map used to make a map of the expected sequence being amplified.

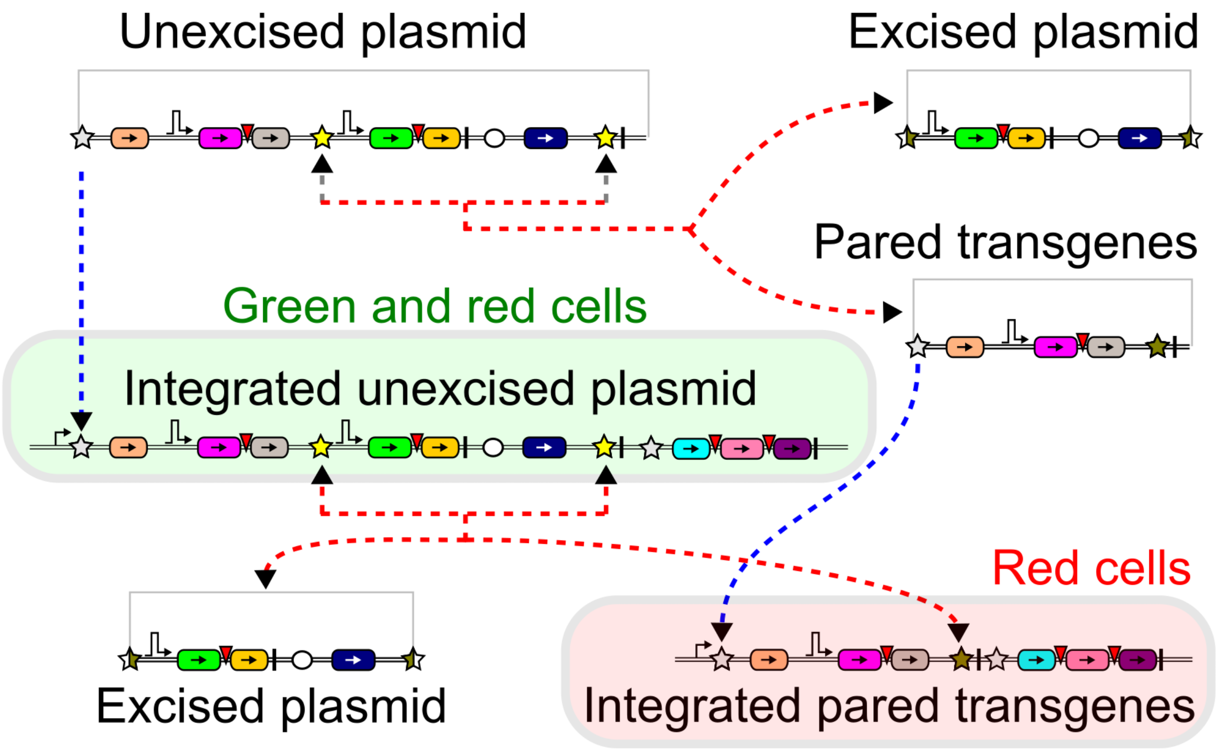

Supplementary Figure S1. Detailed schematic of pared or integrated products possible with the GA attB flanked construct. A more detailed schematic version of the products described in Figure 2B.

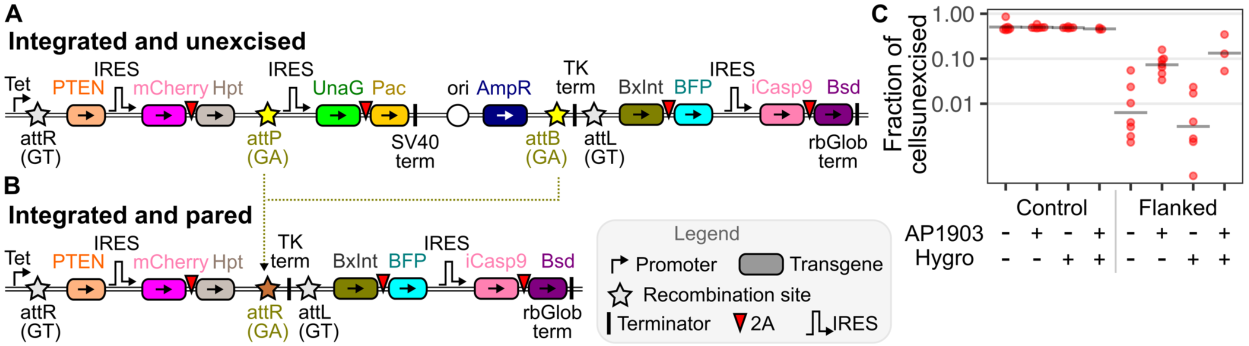

Supplementary Figure S2. Results from integration of the GA-site flanked construct in LLP-Int-iCasp9-Blast cells. Detailed schematic of the A) unexcised or B) pared flanked DNA construct integrated into the LLP-Int-iCasp9-Blast landing pad. C) Of the red cells that formed upon transfection of the control of GA-flanked recombination constructs, the fraction of red cells that were simultaneously green, either in unselected sample or cells treated with AP1903 and/or hygromycin.

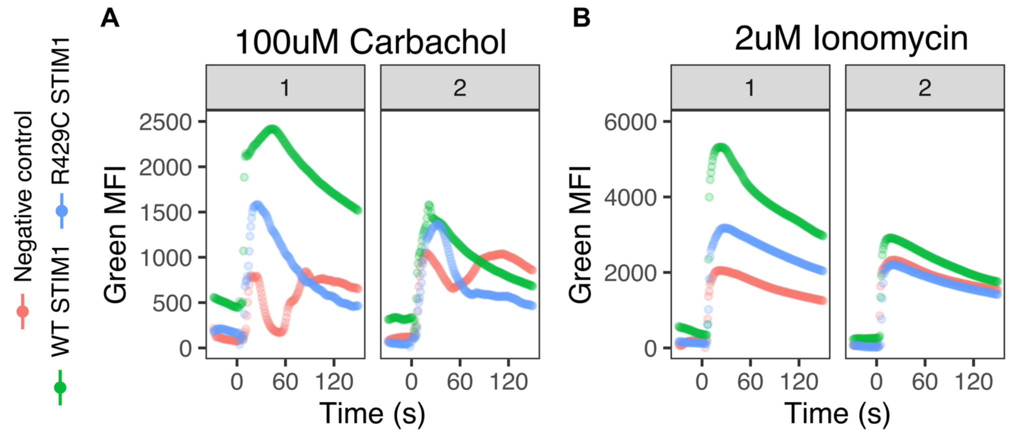

Supplementary Figure S3. Fluorescent intensities of carbachol and ionomycin treated cells without normalization. Raw, unprocessed green fluorescence intensities from two replicate experiments (Numbered 1 and 2), either treated with (A) carbachol or (B) ionomycin at 30 seconds following the start of acquisition (time zero). The red, green, and blue lines correspond to values from cells co-expressing the GCaMP and either the negative control transgene, WT STIM1, or the R429C dominant negative variant, respectively. These values were used to perform the analyses shown in Figure 4.

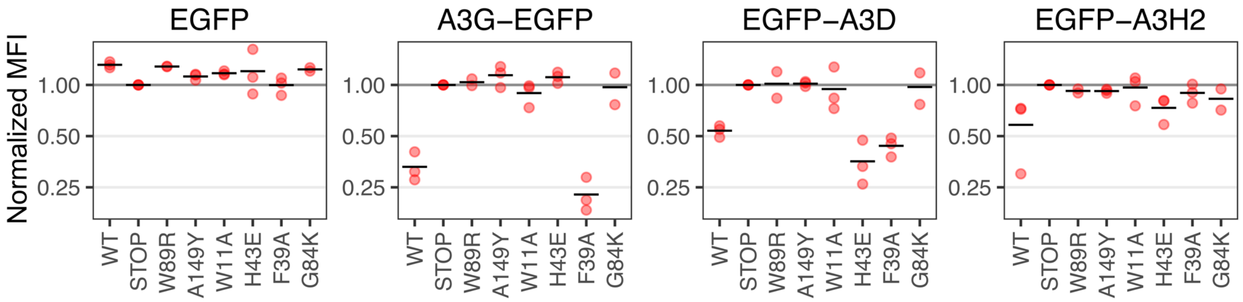

Supplementary Figure S4. Replicate normalized GFP intensity values. The geometric means of green mean fluorescence intensity of each sample in each replicate experiment, normalized to that of the sample with the Vif nonsense mutant (STOP), is shown as red points. The geometric mean of these values across replicate experiments are shown as black bars. These geometric mean values are presented as the heat map in Figure 5.
